# Effects of Osthole on Inflammatory Gene Expression and Cytokine Secretion in Histamine-Induced Inflammation in the Caco-2 Cell Line

**DOI:** 10.3390/ijms222413634

**Published:** 2021-12-20

**Authors:** Natalia K. Kordulewska, Justyna Topa, Dominika Rozmus, Beata Jarmołowska

**Affiliations:** 1 Department of Biochemistry, Faculty of Biology and Biotechnology, University of Warmia and Mazury, 10-719 Olsztyn, Poland; dominika.rozmus@uwm.edu.pl (D.R.); bj58@wp.pl (B.J.); 2 Laboratory of Translational Oncology, Intercollegiate Faculty of Biotechnology, Medical University of Gdansk, 80-211 Gdansk, Poland

**Keywords:** pro-inflammatory cytokine, anti-inflammatory cytokine, interleukin, gene expression, inflammatory intestinal disease

## Abstract

Hyperactivity of the immune system in the gastrointestinal tract leads to the development of chronic, inflammation-associated disorders. Such diseases, including inflammatory bowel disease, are not completely curable, but the specific line of treatment may reduce its symptoms. However, the response to treatment varies among patients, creating a necessity to uncover the pathophysiological basis of immune-mediated diseases and apply novel therapeutic strategies. The present study describes the anti-inflammatory properties of osthole during histamine-induced inflammation in the intestinal Caco-2 cell line. Osthole reduced the secretion of cytokines (CKs) and the expression level of inflammation-associated genes, which were increased after a histamine treatment. We have shown that the secretion of pro-inflammatory CKs (IL-1β, IL-6, IL-8, and TNF-α) during inflammation may be mediated by NFκB, and, after osthole treatment, this signaling pathway was disrupted. Our results suggest a possible role for osthole in the protection against inflammation in the gastrointestinal tract; thus, osthole may be considered as an anti-inflammatory modulator.

## 1. Introduction

Excessive inflammation in the gastrointestinal tract leads to the development of inflammatory bowel disease (IBD), which is a chronic gastrointestinal disorder [1]. The symptoms of the disease can be reduced by using appropriate pharmacological agents, but IBD is not fully curable [2,3,4,5,6]. Two main types of IBD can be distinguished: ulcerative colitis (UC) and Crohn’s disease (CD). UC manifests itself only in the colon as a continuous inflammation that usually begins in the distal colon, progresses through the proximal colon to the appendix, and leads to ulceration and bleeding. Conversely, CD is characterized by patchy lesions in any part of the gastrointestinal tract and is associated with inflammation, stenosis, and/or fistulae [1]. Patients with IBD often receive multiple treatments, and response to the treatment varies widely between patients [7,8], demonstrating the need for a deeper understanding of the disease and the application of innovative therapeutic approaches. Increasing knowledge of the pathophysiology of immune-mediated diseases has led to the development of targeted therapies that can selectively interfere with the crucial mediators of the inflammatory process [9,10,11,12].

Osthole is a natural coumarin-derivative and bioactive compound extracted from medicinal plants, such as *Cnidium monnieri* (L.) Cusson. It has potential therapeutic applications due to its significant and diverse pharmacological properties, including anti-cancer [13,14,15,16], anti-inflammatory [17,18,19], anti-allergic [17,20,21,22,23,24,25], osteoprotective [26], neuroprotective [27,28], and hepatoprotective [29,30,31].

Our previous studies have confirmed that osthole possesses anti-inflammatory properties in peripheral blood mononuclear cells (PBMCs) isolated from children with allergy, autism spectrum disorder (ASD), and adults with allergy and asthma [17,20,21,22,23,24,25]. Osthole prevents lipopolysaccharides (LPS)-induced inflammation in the Caco-2 cell line cultured with THP-1 and THP-1-derived macrophages [32].

In the present study, an in vitro approach is used to evaluate the anti-inflammatory properties of osthole, and its potential use as a drug. In current experiments, the Caco-2 cell line is used as a human epithelium model, because it reflects the morphology and function of human intestinal epithelial cells [33,34,35]. The Caco-2 cell line is widely used to study the induction or inhibition of cells proliferation, cytokines (CKs) secretion, changes in gene expression under the influence of exogenous substances [33,36,37,38], and transepithelial transport of a variety of chemicals, including drugs [39,40].

The effect of osthole has been compared to fexofenadine hydrochloride (FXF), the active metabolite of terfenadine and a third-generation anti-histamine agent, characterized by a poor ability to cross the blood–brain barrier [41].

To induce inflammation in the intestinal model, Caco-2 cells were incubated with histamine [42,43]. We hypothesized that osthole prevents histamine-induced changes in the secretion of pro-(IL-1β, IL-6, IL-8, interferon-gamma—IFN-γ, tumor necrosis factor-alpha—TNF-α) and anti-inflammatory (IL-4, IL-10, IL-13) CKs, and in the expression of genes encoding histamine receptor H1 (*HRH1*), histamine receptor H4 (*HRH4*), interleukin 1 receptor type 1 (*IL1R1*), interleukin 4 receptor (*IL4R*), nuclear factor-kappa B (*NFκB*), and cyclooxygenase-2 (*COX-2*) in the Caco-2 cell line.

## 2. Results

### 2.1. Histamine, Fexofenadine, and Osthole Do Not Affect Cell Viability and Proliferation in Time Point Chosen for Further Analyses

**Figure 1:**
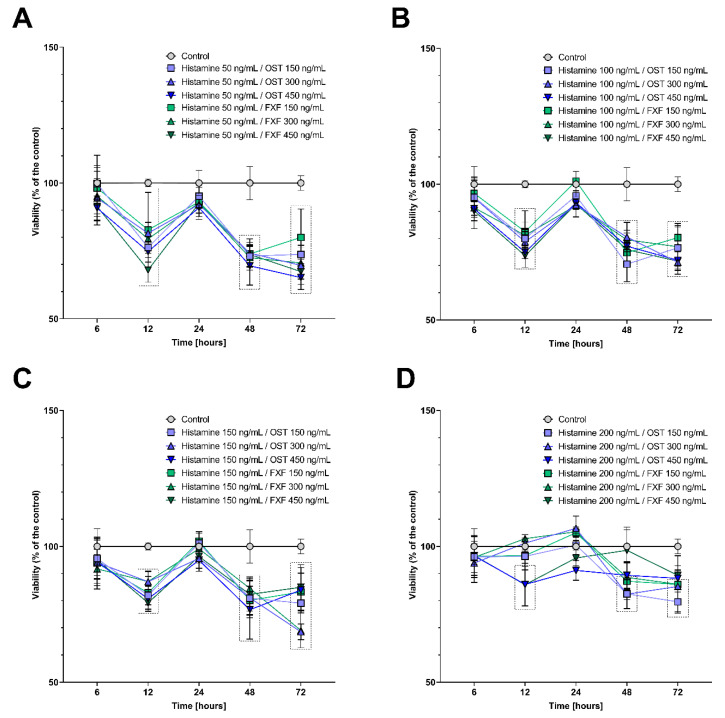
Changes in viability of the Caco-2 cell line after incubation with 50 ng/mL (**A**), 100 ng/mL (**B**), 150 ng/mL (**C**), and 200 ng/mL of histamine (**D**) in mixtures with fexofenadine and osthole (150, 300, and 450 ng/mL). The symbols show the mean and the bars depict the standard deviation. Statistically significant differences compared to control, i.e., cells cultured in medium (*p* < 0.05, two-way ANOVA with Dunnett’s multiple comparisons test) are shown in rectangles with dotted edges. The analyzes were performed in triplicate in three independent experiments.

To select the appropriate time of incubation with histamine, osthole, and FXF in the experiments, cell viability was assessed using a methyl thiazolyl tetrazolium (MTT) assay. To exclude the bias caused by increased cells mortality resulting from the cytotoxic effect of tested substances, Caco-2 cells were incubated with the tested compounds for 24 h. At this time point, the viability of cells incubated with histamine, FXF, and osthole alone (Figure S1) and with mixtures of the substances (Figure 1) did not differ significantly from the control.

**Figure 2:**
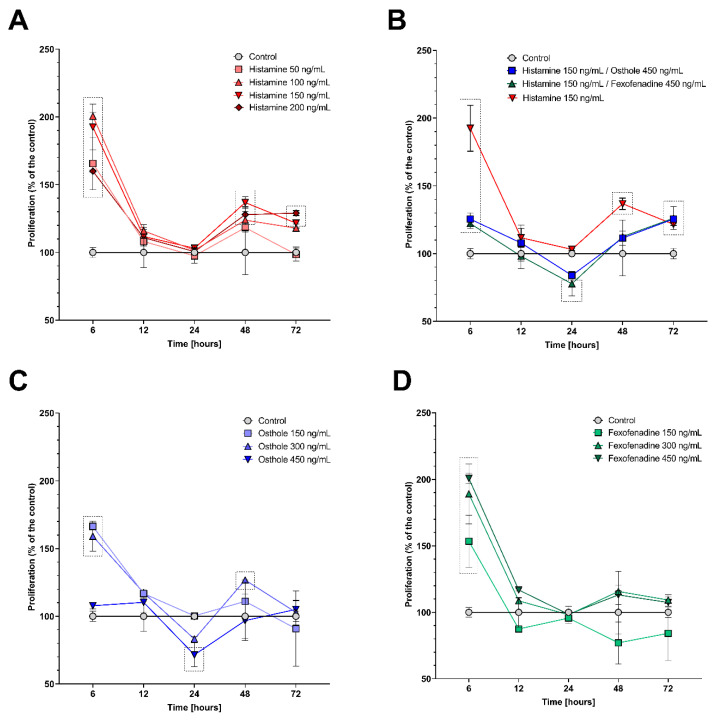
Changes in proliferation of the Caco-2 cell line after incubation with 50 ng/mL, 100 ng/mL, 150 ng/mL, and 200 ng/mL of histamine (**A**); 150 ng/mL, 300 ng/mL, and 450 ng/mL of FXF (**C**); 150 ng/mL, 300 ng/mL and 450 ng/mL of osthole (**D**); and with mixtures of 150 ng/mL of histamine and 450 ng/mL of FXF or osthole (**B**). The symbols show the mean and the bars depict the standard deviation. Statistically significant differences compared to control, i.e., cells cultured in medium (*p* < 0.05, two-way ANOVA with Dunnett’s multiple comparisons test) are shown in rectangles with dotted edges. The analyzes were performed in triplicate in two independent experiments.

To confirm the obtained results, we analyzed cells proliferation by a bromodeoxyuridine (BrdU) incorporation. After 6 h, the proliferation increased significantly for all tested variants (*p* < 0.0001 for all concentrations, except 450 ng/mL of osthole—*p* < 0.05). After 12 h, the proliferation decreased to the level of the control. After 48 h, an increasing trend was observed for most of the tested variants, except 450 ng/mL of FXF, where proliferation dropped, but this change was not significant (Figure 2). A significant increase in proliferation was observed after the incubation with the four histamine concentrations (50 ng/mL—*p* < 0.05, 100 ng/mL—*p* < 0.01, 150 ng/mL—*p* < 0.0001, 200 ng/mL—*p* < 0.001; Figure 2A), and with 300 ng/mL of osthole (*p* < 0.01; Figure 2D). This trend continued until 48 h of incubation with three out of four doses of histamine (100 ng/mL—*p* = 0.01, 150 ng/mL—*p* < 0.01, 200 ng/mL—*p* < 0.0001) and its mixtures with FXF and osthole (*p* < 0.001, for both), where proliferation was significantly higher compared to the control (Figure 2A,B).

### 2.2. Osthole Decreases the Level of Pro-Inflammatory (IL-1β, IL-6, IL-8, TNF-α), but Increases the Level of Anti-Inflammatory CKs (IL-4, IL-10, IL-13) in the Histamine-Treated Caco-2 Cell Line

To determine the effect of osthole and FXF on histamine-induced inflammation in the Caco-2 cell line, we examined the secretion of CKs in a post-culture medium. The level of four out of five pro-inflammatory CKs (IL-β, IL-6, IL-8, TNF-α) increased in a concentration-dependent manner after histamine treatment (Figure S2A). Histamine also significantly increased the IFN-γ level, but no trend was observed. However, the secretion of anti-inflammatory CKs was increased after treatment histamine, but the application of a higher histamine concentration resulted in lower levels of IL-4, IL-10 and IL-13. This finding was the basis for the selection of histamine concentration 200 ng/mL in further experiments. Osthole and FXF increased IL-1β, IL-6, IL-8, and TNF-α in concentrations of 300 and 450 ng/mL (Figure S2A,B). Both osthole and FXF increased the levels of IFN-γ, IL-4, IL-10, and IL-13, and, in the case of anti-inflammatory CKs, this effect depended on the concentration of the substation (Figure S2A,B).

**Figure 3:**
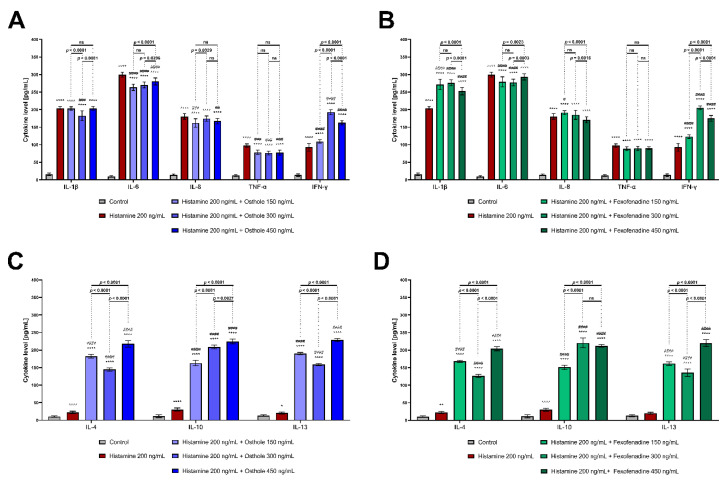
Level of pro-inflammatory (**A**,**B**) and anti-inflammatory (**C**,**D**) CKs after 24 h of incubation with histamine alone and in mixtures with osthole and fexofenadine. The horizontal line shows mean and the bars depict standard deviation. Statistically significant differences (two-way ANOVA with Tukey’s multiple comparisons test) compared to control (cells cultured in medium; *—*p* < 0.05, **—*p* < 0.01, ****—*p* < 0.0001) and to cells treated with histamine (#—*p* < 0.05, ##—*p* < 0.01, ###—*p* < 0.001, ####—*p* < 0.0001) are marked; ns—non-significant. The analyzes were performed in triplicate in three independent experiments.

Osthole significantly decreased the histamine-induced increase in IL-1β (osthole concentration: 300 ng/mL; *p* < 0.0001, Figure 3A), IL-6 (all concentrations; *p* < 0.0001, for both), IL-8 (150 and 450 ng/mL; *p* = 0.0013 and *p* = 0.0029, respectively), and TNF-α (all concentrations; *p* < 0.0001, for all) levels. After incubation with histamine and osthole, the IFN-γ level was elevated (*p* < 0.0001, for all). Surprisingly, FXF, which is a reference drug, did not prevent the increase in IL-8 and TNF-α (Figure 3B). Furthermore, the levels of IL-1β and IFN-γ were even higher when cells were incubated with histamine and FXF, than when treated with histamine alone (*p* < 0.0001, for all). Both osthole and FXF increased the secretion of IL-4, IL-10, and IL-13 (Figure 2C,D; *p* < 0.0001). The levels of pro- and anti-inflammatory CKs, after Caco-2 cell line incubation with lower histamine concentrations and with osthole and FXF, is shown in Figures S3 and S4, respectively.

### 2.3. Osthole at the Lowest Concentration Decreases HRH1, HRH4, IL1R1, IL4R, NFκB, and COX-2 Expression

To understand the essential regulatory mechanisms involved in the regulatory effect of osthole in the Caco-2 cell line under the inflammatory conditions, the expression of *HRH1, HRH4, IL1R1, IL4R, NFκB,* and *COX-2* was analyzed. As expected, histamine increased the expression of *IL1R1*, *NFκB*, and *COX-2* in a concentration-dependent manner (Figure S5A). The *HRH1*, *HRH4* and *IL4R* transcripts were detected only in control and when Caco-2 cells were treated with the highest concentration of histamine. Osthole and FXF also increased the level of the analyzed genes, but the concentration-dependent trend was observed only for *NFκB*, whereas *HRH1*, *HRH4*, and *IL4R* transcripts were not detected (Figure S5B,C).

**Figure 4:**
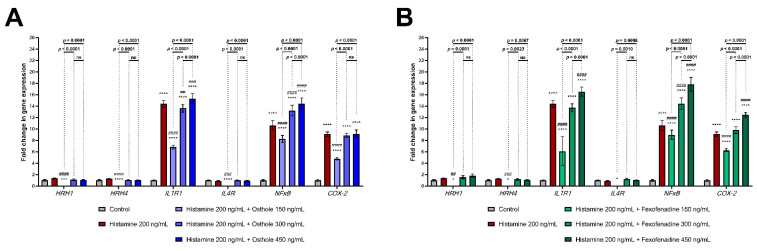
Expression level of *HRH1*, *HRH4*, *IL1R1*, *IL4R*, *NFκB*, and *COX-2* after 24 h of incubation with histamine alone and in mixtures with osthole (**A**) and fexofenadine (**B**). The horizontal line shows mean and the bars depict standard deviation. Statistically significant differences (Two-way ANOVA with Tukey’s multiple comparisons test) compared to control (cells cultured in medium; *—*p* < 0.05, ***—*p* < 0.001,****—*p* < 0.0001) and to cells treated with histamine (##—*p* < 0.01, ###—*p* < 0.001, ####—*p* < 0.0001) are marked; ns—non-significant. The analyzes were performed in triplicate in three independent experiments.

During histamine-induced inflammation, osthole significantly decreased the expression level of *HRH1* (*p* < 0.0001), *HRH4* (*p* < 0.0001), *IL1R1* (*p* < 0.0001), *IL4R* (*p* < 0.0001), *NFκB (p* < 0.0001), and *COX-2* (*p* < 0.0001), but only at the lowest concentration (150 ng/mL; Figure 4A). Higher doses of osthole increased *IL1R1* (450 ng/mL; *p* = 0.0001) expression. Similar effects were observed when Caco-2 cells were treated with histamine and FXF; FXF at the lowest concentration (150 ng/mL) decreased *IL1R1*, *NFκB* and *COX-2* expression, while in the concentration of 450 ng/mL increased the expression of these genes (Figure 4B). The expression level of the examined genes in the Caco-2 cell line after treatment with lower histamine concentrations is shown in Figure S6.

### 2.4. The Secretion of IL-1β, IL-6, IL-8, TNF-α, and the Expression of IL1R1 and COX-2 Are Interdependent in Osthole-Treated Caco-2 with Histamine-Induced Inflammation

**Figure 5:**
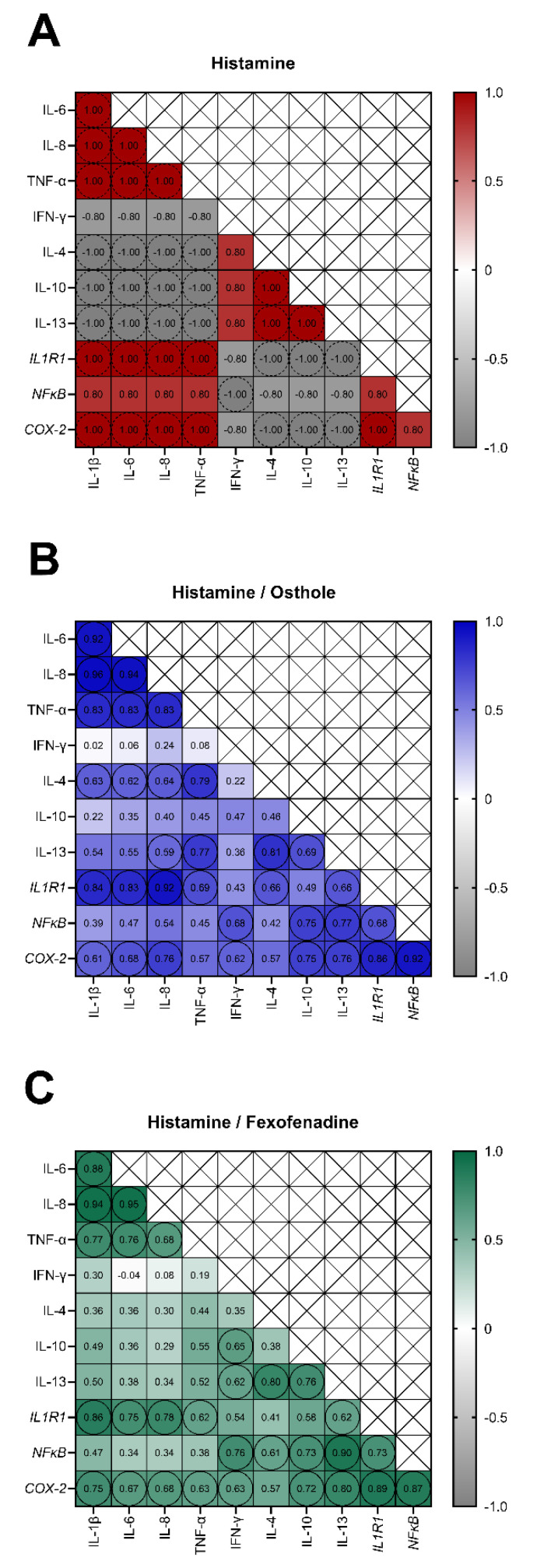
Spearman’s correlations coefficients (r) between cytokines secretion and genes expression level in Caco-2 cells treated with histamine (**A**), histamine and osthole (**B**), and histamine and fexofenadine (**C**) for 24 h. Trends (*p* = 0.083, only in (**A**)) are shown in circles with dotted edges; statistically significant correlations (*p* < 0.05) are marked in circles.

To reveal the links between the expression of the studied cytokines and genes, we performed Spearman’s rank correlation analysis. When we analyzed the correlations after histamine treatment, IL-1β, IL-6, IL-8, TNF-α, *IL1R1* and *COX-2* tended to positively correlate with each other (r = 1.00, *p* = 0.083), and negatively correlate with IL-4, IL-10, and IL-13 (r = −1.00, *p* = 0.083; Figure 5A). The secretion of anti-inflammatory CKs (IL-4, IL-10, and IL-13) also tended to be interdependent (r = 1.00, *p* = 0.083). Conversely, in Caco-2 treated with histamine and osthole, the secretion of IL-1β, IL-6, IL-8, TNF-α, IL-4, IL-13, and the expression of *IL1R1* and *COX-2* were positively correlated (Figure 5B). In cells treated with histamine and FXF, the group of correlating CKs and genes was similar, but lacked the anti-inflammatory IL-4 and IL-13 (Figure 5C).

## 3. Discussion

The aim of this study was to investigate the role of osthole in histamine-induced inflammation in a monolayer of intestinal cells. We hypothesized that osthole may modulate the inflammatory response, as reflected by CK secretion and inflammation-associated genes expression.

The number of patients suffering from inflammation-associated intestinal disorders has increased dramatically in developed countries, and a variety of factors are known to influence this phenomenon [44,45,46]. Therefore, new anti-inflammatory agents need to be investigated and tested as potential therapeutics. In this study, the effects of osthole and FXF on histamine-induced inflammation in the Caco-2 cell line were investigated. The levels of pro- and anti-inflammatory CKs in the post-culture media were measured, and the expression of critical inflammatory genes was analyzed to confirm the posed hypothesis.

Literature data suggest that pro-inflammatory CKs activate noradrenergic transmission and the hypothalamic–pituitary–adrenal (HPA) axis, and cause glucocorticoid receptor resistance [47,48]. FXF, a third-generation anti-histamine, has been applied as a positive control, as it does not attenuate the presence and function of histamine H1 and glucocorticoid receptors [47,49]. The discovery of the relationship between the intestinal barrier, the activation of the inflammatory response (CKs secretion, changes in gene expression), and the action of osthole and FXF may provide the basis for improving therapeutic schemes for intestinal inflammation.

The incubation time was selected based on Caco-2 cells viability during incubation with the tested compounds. While cell viability, mostly, was not affected by the tested substances (Figure 1 and Figure S1), cell proliferation after treatment with histamine (50–200 ng/mL), FXF, and osthole (150–450 ng/mL, for both) caused a significant increase in proliferation after 6 h of the experiment (Figure 2). After 6 h, fluctuations in cells viability measured by MTT assay were also observed; histamine and fexofenadine mostly increased cell viability, while osthole decreased it. These changes might have been caused by stressful conditions, such as the addition of exogenous substances to the culture medium, especially that histamine is a well-known agent stimulating colorectal cell line proliferation, including Caco-2 [50,51].

At subsequent time points, FXF and osthole did not significantly increase cell proliferation (with the exception of 300 ng/mL osthole at 48 h). Interestingly, cell proliferation was not significantly different from control after 12 h and 24 h of incubation with histamine, but it was again increased after 48 h and 72 h. We speculate that this might be caused due to a renewed cellular response to the presence of an inflammatory factor.

In our experiments, incubation of Caco-2 with histamine resulted in a marked increase in secretion of all tested CKs. According to our knowledge, this is the first study investigating the effects of histamine on the secretion of the tested CKs in Caco-2 cells. Lügering et al. described that the stimulation of Caco-2 with IL-1β caused a time-dependent increase in IL-8 secretion [52]. In our study, IL-1β and IL-8 were increased after incubation with histamine (Figure S2A), and the secretion of these CKs was correlated (Figure 5A). It is worth to underline that osthole significantly decreased the secretion of IL-1β, IL-6, IL-8, and TNF-α in the histamine-induced Caco-2 cell line, especially at the lower concentrations (150–300 ng/mL; Figure 3B), suggesting that the therapeutic effect is observed only after treatment with certain osthole concentrations. The effect of osthole was compared with FXF, an anti-histamine drug that has been shown to prevent inflammatory responses in mice with induced colitis [53,54]. Surprisingly, our results showed that osthole has a higher potential than FXF to decrease the histamine-induced secretion of pro-inflammatory CKs, thus FXF should not be considered as a positive control in this experiment.

**Figure 6:**
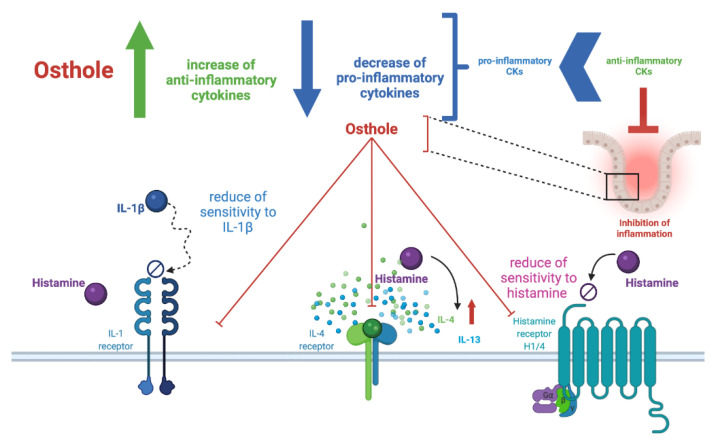
Osthole regulates inflammatory responses by shifting the balance in pro-inflammatory and anti-inflammatory CK secretion, which results in a reduction in the sensitivity to histamine and other pro-inflammatory agents.

Lügering et al. showed that the addition of IL-4 and -13, but not IL-10, caused a significant decrease in IL-8 secretion [52]. In our study, the secretion of IL-4, IL-10, and IL-13 tended to negatively correlate with the IL-8 secretion, partially confirming the results described by Lügering et al. IL-8 plays an important role in acute and chronic processes, while IL-4 and-13 are down regulated in the inflammatory response [52]. However, in our experiment, the incubation of the Caco-2 cell line with histamine resulted in an increase in the secretion of IL-4, IL-10, and IL-13 (Figure S2), and, after the addition of osthole or FXF, the level of these CKs was even higher (Figure 3C,D). The ratio in the secretion of pro- and anti-inflammatory CKs determines local inflammation (Figure 6). Osthole, as a substance with demonstrated anti-inflammatory properties [55], increased the secretion of the anti-inflammatory IL-4, IL-10, and IL-13 under normal (cells treated only with osthole; Figure S5B) and inflammatory conditions (cells treated with histamine and osthole; Figure 3C). The protective role of osthole was demonstrated in rats with induced ischemia, which caused an increase in plasma pro-inflammatory CKs level and a decrease in anti-inflammatory CKs in the infarct area [56].

High concentrations of IL-4/IL-13 and IL-10 in the microenvironment may alter immune cell recruitment to enterocytes, at least in part by reducing IL-8 secretion. Such inhibition may reduce the severity of the intestinal inflammatory responses and reduce clinical disease activity [57]. IL-10 counteracts the influence of pro-inflammatory CKs on the barrier properties of the epithelium or endothelium [58,59]. Mazzon et al. observed that IL-10 knockout mice, a model of spontaneous colitis, have increased levels of pro-inflammatory TNF-α, IL-1β, and IL-6 [60].

In addition to influencing the secretion of CKs, osthole also altered the expression of genes encoding interleukin receptors (Figure 6). During histamine-induced inflammation, the decrease in IL-1β secretion after the treatment with osthole correlated with a decreased expression of *IL1R1*, leading to the conclusion that osthole affects both IL-1β secretion and Caco-2 cells sensitivity to this CK.

We observed that histamine significantly increased *HRH1* expression level, while osthole and FXF (at the lowest concentration) reduced this effect (Figure 4), indicating the role of osthole in reducing the sensitivity of cells to histamine (Figure 6). Giustizieri et al. evaluated the stimulatory effect of histamine on *HRH1* expression in keratinocytes and showed that levocetirizine (an anti-histamine drug) inhibited histamine-induced secretion of inflammatory CKs in a dose-dependent manner. HRH1 was shown to activate NFκB [61]. Since both HRH1 and NFκB are involved in inflammation, we can speculate that the link between HRH1 and NFκB is physiologically important in inflammation-associated diseases. Bakker et al. demonstrated that NFκB activation via HRH1 is inhibited by several anti-histamine drugs, including cetirizine, ebastine, epinastine, FXF, loratadine, and mizolastine [62]. Our results support this finding, as both osthole and FXF reduced the histamine-induced increase in *NFκB* expression (Figure 4). Moreover, both osthole and FXF at lowest concentration reduced the overexpression of *COX*-2 after histamine treatment. Similar results were obtained by Yang et al., who found that the expression of *COX-2* was downregulated after stimulation with a mixed formula of Chinese herbs [63].

The pathogenesis of intestinal inflammation is a complex process involving alterations in intestinal barrier function and food intolerances, leading to activation of the innate immune system. However, there is evidence that NFκB activation in mucosal epithelia is a crucial event in this process [64,65]. Anti-inflammatory therapies, such as anti-TNF-α antibodies and steroids that regulate NFκB activation, are commonly used for IBD treatment, but are associated with side effects [66,67]. Although a modified diet and certain nutrients have been shown to attenuate intestinal inflammation, it is unclear whether they act by altering the microbiome, innate immunity, and/or cellular response to inflammation [68,69]. NFκB plays a central role in the connection between external pro-inflammatory stimuli and the expression of inflammatory genes [70] and can be also activated by pro-inflammatory agents, such as IL-1α, IL-1β, and TNF-α [71,72]. Therefore, the disruption of NFκB activation may be particularly beneficial in diseases related to chronic, low-grade inflammation [73]. IL-1β is involved in the pathogenesis of intestinal inflammation and stimulates NFκB activation [72] and, after histamine stimulation, increased levels of IL-1β and expression of *NFκB* were observed. Therefore, we propose that histamine-induced IL-1β secretion and *NFκB* expression are interdependent, especially as IL-1β secretion and *NFκB* expression tended to correlate with each other (Figure 5A). Although osthole at the lowest concentration effectively decreased IL-1β and *NFκB* levels, we speculate that osthole might decrease IL-1β-induced *NFκB* activation. We also speculate that NFκB inhibition and slight response in IL-1β secretion might be related to the regulation of Nod-like receptor (NLR) family pyrin domain containing protein 3 (NLRP3) inflammasome regulation [74], which belongs to a group of multiprotein complexes within cells formed in response to pathogen-associated (PAMPs) and damage-associated molecular patterns (DAMPs) [75]. Canonical inflammasomes consist of a ligand-responsive receptor (e.g., NLR family member), the adaptor protein ASC (apoptosis-associated speck-like protein containing CARD), and pro-caspase-1 [76]. Upon stimulation, the inflammasome receptor oligomerizes and recruits pro-caspase-1 via the ASC, which stimulates the processing and conversion of pro-caspase 1 into active caspase 1. Activated caspase 1 cleaves pro-IL-1β and pro-IL-18 into their mature forms, resulting in the release of these pro-inflammatory CKs [76]. We suspect that Caco-2 cells experience an increase in pro-IL-1β cleavage in response to stimulation by histamine and/or histamine + osthole, in relation to control, leading to an increased secretion of the active IL-1β form.

Our hypothesis is also supported by the fact that the NFκB pathway is involved in the regulation of inflammasomes and contributes to the onset and development of inflammatory diseases [77,78,79]. Based on the obtained results, we suspect that, similar to glucocorticoids and peroxisome proliferator-activated receptor (PPAR) agonists [80], osthole prevents NFκB subunits binding to target genes and inhibits transcription without having a clear effect on CKs secretion.

We demonstrated that Caco-2 cells secrete IL-8 after incubation with histamine, osthole, and FXF in a concentration-dependent manner. In the Caco-2 cell line, secretion of IL-8 can be induced by IL-1β via the NFκB pathway [81]. Hoffman et al. demonstrated a positive correlation between IL-8 and *NFκB*, which was also found in our study after Caco-2 incubation with histamine (Figure 4A), and may explain the inhibitory effect of osthole on IL-8 secretion [82].

In this study, inflammation in the Caco-2 cell line was generated by histamine stimulation. We are aware that a simplified in vitro model does not fully reflect the complex architecture and organization of the intestine, which may affect the translation of our results to the processes occurring in the organism. Unfortunately, we are still far from being able to fully replace studies on human tissues or animal models with in vitro systems. These limitations can only be overcome if all relevant mechanisms of the investigated inflammatory reactions are known.

## 4. Materials and Methods

### 4.1. Chemicals

Osthole (PubChem CID: 10228), histamine (PubChem CID: 774), and FXF (PubChem CID: 63002) were obtained from Sigma Aldrich (St. Louis, MO, USA, cat. no. O9265, H7125, F9427, respectively). Osthole was dissolved in 96% ethanol (Chempur, Piekary Śląskie, Poland, cat. no. 653964200); FXF was dissolved in 8% dimethyl sulfoxide (DMSO; Sigma Aldrich, St. Louis, MO, USA, cat. no. D2650); and histamine was dissolved in the medium used for cell culture. The solutions were filtered through 0.22 µm pore filters and stored at −20 °C for later dilution.

### 4.2. Cell Culture

A Caco-2 cell line obtained from the American Tissue Culture Collection (ATCC, Manassas, VA, USA) was maintained in T-75 flasks in Dulbecco’s Modified Eagle’s Medium (DMEM; Sigma-Aldrich, St. Louis, MO, USA, cat. no. D6429), and supplemented with 10% fetal bovine serum (Gibco, Thermo Fisher Scientific, Waltham, MA, USA, cat no. 16000044), 1% non-essential amino acids serum (Gibco, Thermo Fisher Scientific, Waltham, MA, USA, cat. no. 11140050), 0.5% penicillin/streptomycin (Sigma Aldrich, St. Louis, MO, USA, cat. no. P4333), and 0.1% of gentamicin (Sigma Aldrich, St. Louis, MO, USA, cat. no. G1272). The cells were incubated at 37 °C in a 95% humidified atmosphere and 5% CO_2_. The culture medium was changed every 2–3 days, and when a confluence of 80–90% was reached, the cells were passaged.

### 4.3. Cells Viability Analysis

Cell viability was analyzed using MTT Cell Proliferation Assay Kit (Abcam, Cambridge, UK, cat. no. ab211091) according to the manufacturer’s protocol. Briefly, cells were seeded in the culture medium at concentration of 1 × 10^4^ cells per well in 96-well plates. After 24 h, the medium was removed and replaced with the medium with tested compounds. The cells were incubated for 6 h, 12 h, 24 h, 48 h, and 72 h. After incubation, plates were spined at 1000× *g*, 4 °C for 5 min and media were aspirated. To each well, 50 µL of a serum-free medium and 50 µL of MTT Reagent were added, and the plates were incubated at 37 °C for 3 h. After that time, 150 µL of MTT solvent was added to each well, and plates were incubated on a shaker at 37 °C in the dark. Absorbance was read at 590 nm. The percent of viable cells was calculated in reference to control (100%, cells seeded in DMEM).

### 4.4. Cells Proliferation Analysis

Proliferation of Caco-2 cells in the presence of various concentrations of tested compounds was examined using the Cell Proliferation ELISA, BrdU (colorimetric) Kit (Roche Diagnostics, Basel, Switzerland, cat. no. 11647229001). Cells were seeded in the culture medium in 96-well plates at a concentration of 5 × 10^3^ cells per well. After 24 h, the medium was removed and replaced with the medium containing tested substances and BrdU in the final concentration of 10 µM. The cells were incubated for 6 h, 12 h, 24 h, 48 h, and 72 h. After incubation, the medium was removed by flicking off and the cells were dried at 60 °C for 1 h. the plates were coated with parafilm and stored at 4 °C for up to 3 days. The proliferation was analyzed according to the manufacturer’s instructions. The percent of cells was calculated in reference to the control (100%, cells seeded in DMEM).

### 4.5. Incubation of Caco-2 Cells with Examined Substances

The cells were seeded at a concentration of 2.5 × 10^4^ cells per well in a culture medium in 24-well plates. The medium was removed after 24 h and replaced with fresh DMEM with the addition of tested substances in the final volume of 1 mL. After 24 h of culture, the medium was collected, and the total RNA was isolated and reverse transcribed.

### 4.6. Post-Culture Media Collection and Total RNA Isolation

The cell culture plates were centrifuged at 4 °C, 800× *g* for 10 min, and post-culture media were collected in tubes and stored immediately at −80 °C for further analysis. Cells were lysed with 1 mL of TRIzol™ Reagent (Invitrogen, Thermo Fisher Scientific, Waltham, MA, USA, cat. no. 15596026), and, after 5 min of incubation at room temperature, 0.2 mL of chloroform (Chempur, Piekary Śląskie, Poland, cat. no. 112344305) was added. All samples were mixed and centrifuged at 4 °C, 12,000× *g* for 15 min. The aqueous phase was collected and mixed with 0.5 mL of isopropanol to precipitate the RNA. The supernatant was discarded, and the RNA pellet was washed with 75% ethanol. The RNA pellet was air dried and dissolved in diethyl pyrocarbonate (DEPC)-treated water. The absorbance at 260 nm and 280 nm (A_260_/A_280_) was measured to determine the amount and purity of RNA.

### 4.7. Reverse Transcription and Quantitative Real-Time PCR (qPCR)

Purified RNA was reverse transcribed using the High-Capacity cDNA Reverse Transcription Kit (Applied Biosystems, Thermo Fisher Scientific, Waltham, MA, USA, cat. no. 4368814), according to the manufacturer’s protocol. The cDNA was stored at −20 °C for further analysis.

Changes in the expression level of *HRH1*, *HRH4*, *IL1R1*, *IL4R*, *NFκB*, *COX-2,* and actin beta (*ACTB*) were examined. *ACTB* was used as a reference gene to normalize disproportion in the mRNA amount. Oligonucleotide primers specific to each gene are listed in Table S1. The qPCR was performed in the LightCycler 96 Real-Time PCR System using the FastStart Essential DNA Green Master Kit (Roche Diagnostics, Basel, Switzerland, cat. no. 06402712001), according to Kordulewska et al. [25]. Samples were run in triplicates, and 5 ng of cDNA was used for each reaction. Negative control samples without cDNA and an inter-run calibrator were included in each assay. The qPCR was performed under the following conditions: denaturation at 95 °C for 10 min, amplification (20 s at 95 °C, 20s at 60/62/63 °C) and quantification (20 s at 72 °C with a single fluorescence measurement) repeated 45 times, melting curve at 60–95°C with a heating rate of 0.1 °C per second and continuous fluorescence measurement and final cooling down to 4 °C. Gene expression was analyzed according to Pfaffl [83], and the results were scaled to the expression of the control, which was determined as 1.

### 4.8. Analysis of Cytokines Level

Enzyme-linked immunosorbent assay (ELISA) kits were used to measure the levels of IL-1β, -4, -6, -8, -10, -13, IFN-γ, and TNF-α in the post-culture medium. Kits were obtained from Diaclone (Besancon Cedex, France; TNF-α—cat. no. 851.570.001, IL-10—cat. no. 851.540.001, IL-1β—cat. no. 851.610.001,), Mabtech (Nacka Strand, Sweden; IFN-γ—cat. no. 3420-1H-6, IL-13—cat. no. 3470-1H-20, IL-6—cat. no. 3460-1H-20, IL-4—cat. no. 3410-1H-20), and BD Biosciences (San Jose, CA, USA; IL-8—cat. no. 555244). All kits were used according to the manufacturer’s protocols. The samples were run in quadruples. The results were compared to a standard curve and standardized.

### 4.9. Statistical Analysis

To show differences between quantitative values, an ordinary two-way ANOVA followed by Dunnett’s or Tukey’s multiple comparison tests were performed. The correlations were examined using Spearman’s rank order test. The statistical significance level was set at a *p*-value of 0.05. GraphPad Prism software version 9.3.1 (GraphPad Software, San Diego, CA, USA) was used for statistical calculations and data visualization.

## 5. Conclusions

Osthole and FXF inhibited IL-1β-mediated inflammatory responses in Caco-2 cells, including the secretion of IL-8 and NFκB activation. While the mechanisms responsible for the observed effects remain to be elucidated, the anti-inflammatory properties of osthole may play an important role. Our data confirmed the potential role of osthole in protection against inflammation in the gastrointestinal tract; therefore, osthole may be considered as an anti-inflammatory modulator in the treatment of inflammation after extensive in vitro and in vivo studies.

## Data Availability

The data presented in this study are available on request from the corresponding author.

## Supplementary Materials

The following are available online at https://www.mdpi.com/article/10.3390/ijms222413634/s1.

## Abbreviations

ACTB	Actin beta
ASC	Apoptosis-associated speck-like protein containing CARD
ASD	Autism spectrum disorder
BrdU	Bromodeoxyuridine
CD	Crohn’s disease
CK	Cytokine
COX-2	Cyclooxygenase 2
DAMPs	Damage-associated molecular patterns
DEPC	Diethyl pyrocarbonate
DMEM	Dulbecco’s Modified Eagle’s Medium
DMSO	Dimethyl sulfoxide
ELISA	Enzyme-linked immunosorbent assay
FXF	Fexofenadine hydrochloride
HPA axis	Hypothalamic-pituitary-adrenal axis
HRH1	Histamine receptor H1
HRH4	Histamine receptor H4
IBD	Inflammatory bowel disease
IFN-γ	Interferon-gamma
IL1R1	Interleukin 1 receptor type 1
IL4R	Interleukin 4 receptor
LPS	Lipopolysaccharides
MTT	Methyl thiazolyl tetrazolium
NFκB	Nuclear factor-kappa B
NLR	Nod-like receptor
NLRP3	NLR family pyrin domain containing protein 3
PAMPs	Pathogen-associated molecular patterns
PBMCs	Peripheral blood mononuclear cells
PPAR	Peroxisome proliferator-activated receptor
TNF-α	Tumor necrosis factor-alpha
UC	Ulcerative colitis
