# Supplementary material for: Effects of Osthole on Inflammatory Gene Expression and Cytokine Secretion in Histamine-Induced Inflammation in the Caco-2 Cell Line
Source: Int J Mol Sci. 2021 Dec 20;22(24):13634. doi: 10.3390/ijms222413634 (PMC8708099; doi:10.3390/ijms222413634)
Supplement: Supplementary file 1 [file ijms-22-13634-s001.zip › ijms-1476186-supplementary/Supplementary Figures/Supplementary Caption.pdf]

Table S1. Sequences of the oligonucleotide primers specific to examined genes

Figure S1. Changes in viability of the Caco-2 cell line after incubation with histamine (**A**), osthole (**B**) and fexofenadine (**C**).The symbols show the mean and the bars depict the standard deviation. Statistically significant differences compared to control – cells cultured in medium ($p < 0.05$, Two-way ANOVA with Dunnet's multiple comparisons test), are shown in rectangles with dotted edges. Analyzes were performed in triplicate in three independent experiments

Figure S2. Level of pro- and anti-inflammatory CKs after 24 h of incubation with histamine **(A)**, osthole **(B)** and fexofenadine **(C)**. The horizontal line shows mean and the bars depict standard deviation. Statistically significant differences (Two-way ANOVA with Tukey's multiple comparisons test) compared to control (* - $p < 0.05$,** - $p < 0.01$, *** - $p < 0.001$, **** - $p < 0.0001$) are marked

Figure S3. Level of pro-inflammatory CKs after incubation with histamine (50, 100, and 150 ng/mL) alone and in mixtures with osthole **(A,C,E)** and fexofenadine **(B,D,F)** for 24 h. The horizontal line shows mean and the bars depict standard deviation. Statistically significant differences (Two-way ANOVA with Tukey's multiple comparisons test) compared to control (** - $p < 0.01$, *** - $p < 0.001$, **** - $p < 0.0001$) and to cells treated with histamine (# - $p < 0.05$, ## - $p < 0.01$, ### - $p < 0.001$, #### - $p < 0.0001$) are marked; ns – non-significant

Figure S4. Level of anti-inflammatory CKs after incubation with histamine (50, 100, and 150 ng/mL) alone and in mixtures with osthole **(A,C,E)** and fexofenadine **(B,D,F)** for 24 h. The horizontal line shows mean and the bars depict standard deviation. Statistically significant differences (Two-way ANOVA with Tukey's multiple comparisons test) compared to control (**** - $p < 0.0001$) and to cells treated with histamine (#### - $p < 0.0001$) are marked; ns – non-significant

Figure S5. Expression level of *HRH1*, *HRH4*, *IL1R1*, *IL4R*, *NFκB*, and *COX-2* after 24 h of incubation with histamine **(A)** osthole (**B**) and fexofenadine **(C)**. The horizontal line shows mean and the bars depict standard deviation. Statistically significant differences (Two-way ANOVA with Tukey's multiple comparisons test) compared to control (*** - $p < 0.001$, **** - $p < 0.0001$) are marked

Figure S6. Expression level of *IL1R1*, *NFκB*, and *COX-2* after incubation with histamine (50, 100, and 150 ng/mL) alone and in mixtures with osthole **(A,C,E)** and fexofenadine **(B,D,F)** for 24 h. The horizontal line shows mean and the bars depict standard deviation. Statistically significant differences (Two-way ANOVA with Tukey's multiple comparisons test) compared to control (**** - $p < 0.0001$) and to cells treated with histamine (# - $p < 0.05$, ## - $p < 0.01$, ### - $p < 0.001$, #### - $p < 0.0001$) are marked; ns – non-significant.
