# Supplementary material for: Effects of Osthole on Inflammatory Gene Expression and Cytokine Secretion in Histamine-Induced Inflammation in the Caco-2 Cell Line
Source: Int J Mol Sci. 2021 Dec 20;22(24):13634. doi: 10.3390/ijms222413634 (PMC8708099; doi:10.3390/ijms222413634)
Supplement: Supplementary file 1 [file ijms-22-13634-s001.zip › ijms-1476186-supplementary/Table S1.pdf]

Table S1. Sequences of the oligonucleotide primers specific to examined genes.

Gene	Sequence	Product lenght (bp)
ACTB	F: 5′-TCCCTGGAGAAGAGCTACGA-3′<br>R: 5′-AGCACTGTGTTGGCGTACAG-3′	194
HRH1	F: 5′-GCCGAGAGGACAAGTGTGA-3'<br>R: 5′-GGAGACTCCTTCCCTGGTTT-3′	244
HRH4	F: 5-′ACTCTGATGGTGGCCGTTT-3′<br>R: 5′-TCAGTCCAGGATGGCTTTG-3′	250
IL1R1	F: 5′-GACAGGGCCTAGCTTTCATTT-3'<br>R: 5′-TGGCCAATTTTGTCACTAACC-3′	204
IL4R	F: 5′-TCATGGATGACGTGGTCAGT-3′<br>R: 5-′GTGTCGGAGACATTGGTGTG-3′	148
NFκB	F: 5′-TGGAGTCTGGGAAGGATTTG-3'<br>R: 5′-CGAAGCTGGACAAACACAGA-3′	129
COX-2	F: 5′-TGGCTACAAAAGCTGGGAAG-3'<br>R: 5′-GCTGCTTTTTACCTTTGACACC-3′	110
