# Supplementary figures and images for: Effects of Osthole on Inflammatory Gene Expression and Cytokine Secretion in Histamine-Induced Inflammation in the Caco-2 Cell Line
Source: Int J Mol Sci. 2021 Dec 20;22(24):13634. doi: 10.3390/ijms222413634 (PMC8708099; doi:10.3390/ijms222413634)

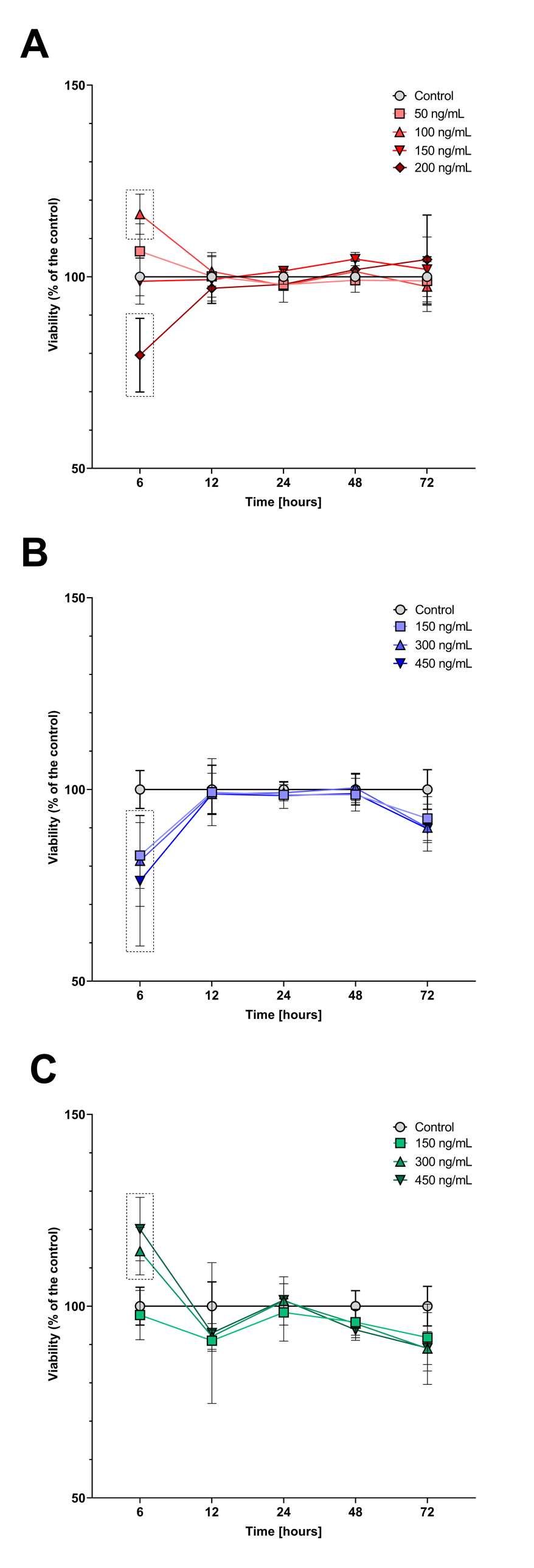

Supplement: Supplementary file 1 [file ijms-22-13634-s001.zip › ijms-1476186-supplementary/Supplementary Figures/Figure S1.png]

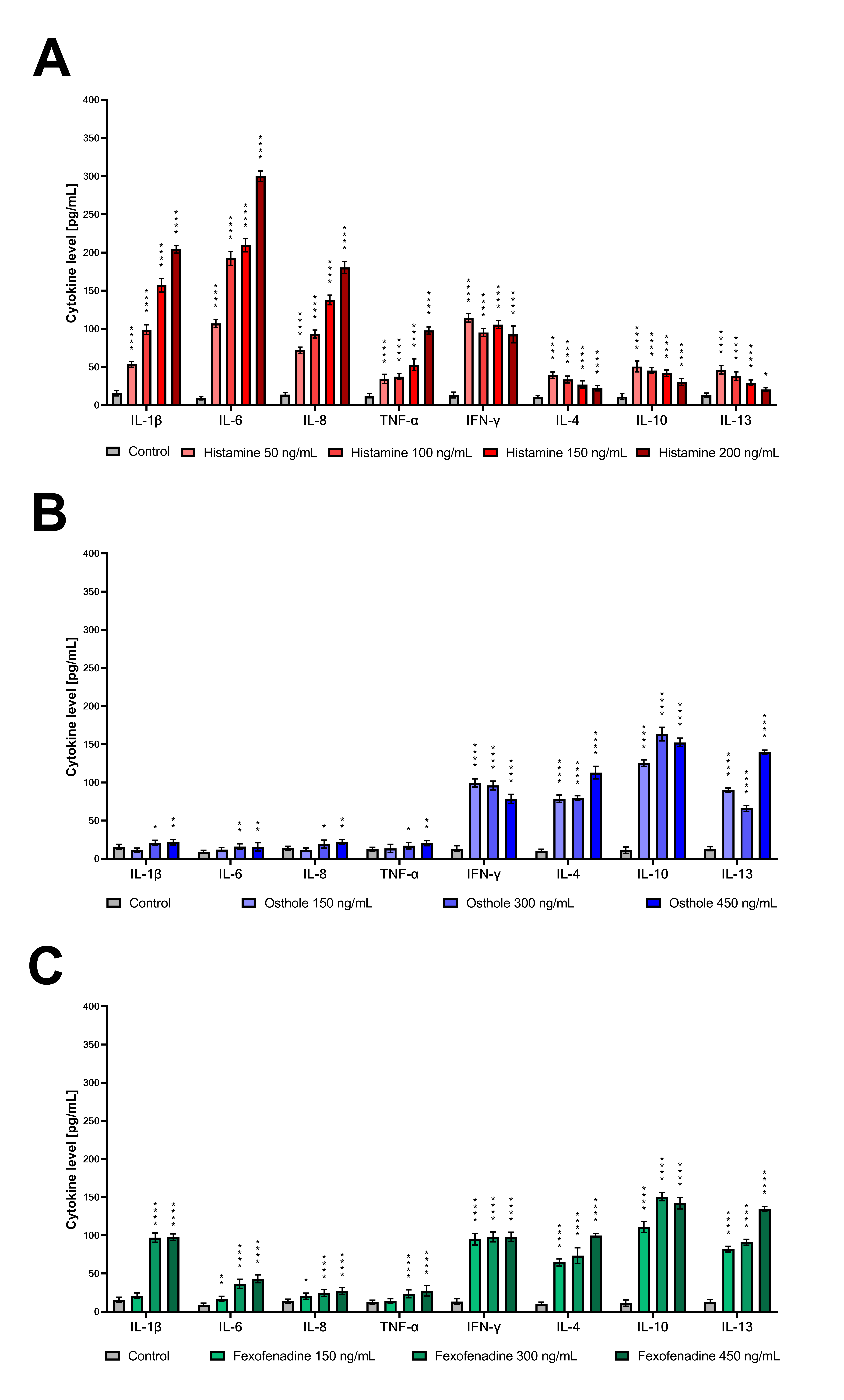

Supplement: Supplementary file 1 [file ijms-22-13634-s001.zip › ijms-1476186-supplementary/Supplementary Figures/Figure S2.png]

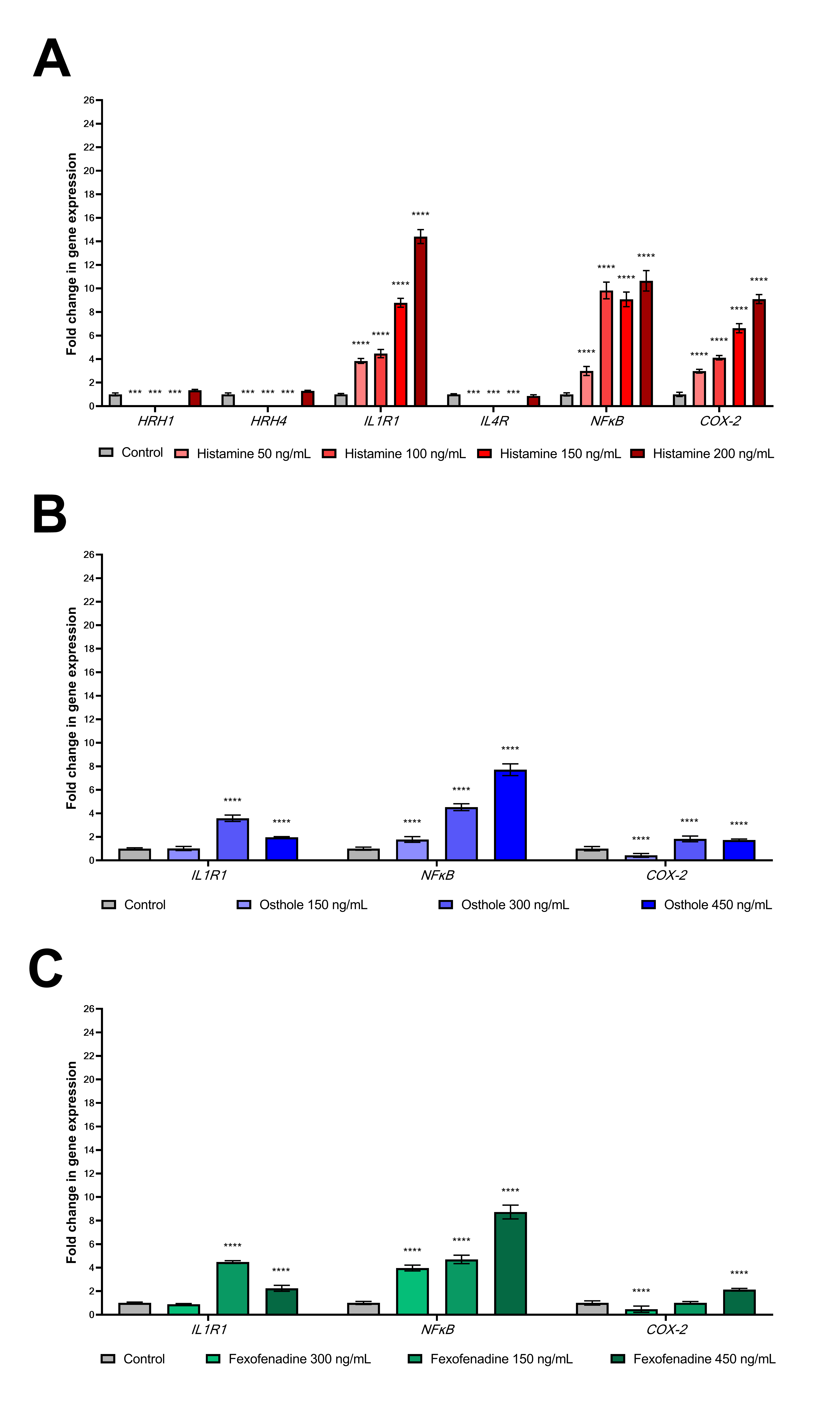

Supplement: Supplementary file 1 [file ijms-22-13634-s001.zip › ijms-1476186-supplementary/Supplementary Figures/Figure S5.png]
